# Risk‐mitigating behaviours in people with inflammatory skin and joint disease during the COVID‐19 pandemic differ by treatment type: a cross‐sectional patient survey^*^

**DOI:** 10.1111/bjd.19755

**Published:** 2021-07-01

**Authors:** S.K. Mahil, M. Yates, S.M. Langan, Z.Z.N. Yiu, T. Tsakok, N. Dand, K.J. Mason, H. McAteer, F. Meynell, B. Coker, A. Vincent, D. Urmston, A. Vesty, J. Kelly, C. Lancelot, L. Moorhead, H. Bachelez, I.N. Bruce, F. Capon, C.R. Contreras, A.P. Cope, C. De La Cruz, P. Di Meglio, P. Gisondi, K. Hyrich, D. Jullien, J. Lambert, H. Marzo‐Ortega, I. McInnes, L. Naldi, S. Norton, L. Puig, R. Sengupta, P. Spuls, T. Torres, R.B. Warren, H. Waweru, J. Weinman, C.E.M. Griffiths, J.N. Barker, M.A. Brown, J.B. Galloway, C.H. Smith, on behalf of the PsoProtect, CORE‐UK study groups

## Abstract

**Background:**

Registry data suggest that people with immune‐mediated inflammatory diseases (IMIDs) receiving targeted systemic therapies have fewer adverse coronavirus disease 2019 (COVID‐19) outcomes compared with patients receiving no systemic treatments.

**Objectives:**

We used international patient survey data to explore the hypothesis that greater risk‐mitigating behaviour in those receiving targeted therapies may account, at least in part, for this observation.

**Methods:**

Online surveys were completed by individuals with psoriasis (globally) or rheumatic and musculoskeletal diseases (RMDs) (UK only) between 4 May and 7 September 2020. We used multiple logistic regression to assess the association between treatment type and risk‐mitigating behaviour, adjusting for clinical and demographic characteristics. We characterized international variation in a mixed‐effects model.

**Results:**

Of 3720 participants (2869 psoriasis, 851 RMDs) from 74 countries, 2262 (60·8%) reported the most stringent risk‐mitigating behaviour (classified here under the umbrella term ‘shielding’). A greater proportion of those receiving targeted therapies (biologics and Janus Kinase inhibitors) reported shielding compared with those receiving no systemic therapy [adjusted odds ratio (OR) 1·63, 95% confidence interval (CI) 1·35–1·97]. The association between targeted therapy and shielding was preserved when standard systemic therapy was used as the reference group (OR 1·39, 95% CI 1·23–1·56). Shielding was associated with established risk factors for severe COVID‐19 [male sex (OR 1·14, 95% CI 1·05–1·24), obesity (OR 1·37, 95% CI 1·23–1·54), comorbidity burden (OR 1·43, 95% CI 1·15–1·78)], a primary indication of RMDs (OR 1·37, 95% CI 1·27–1·48) and a positive anxiety or depression screen (OR 1·57, 95% CI 1·36–1·80). Modest differences in the proportion shielding were observed across nations.

**Conclusions:**

Greater risk‐mitigating behaviour among people with IMIDs receiving targeted therapies may contribute to the reported lower risk of adverse COVID‐19 outcomes. The behaviour variation across treatment groups, IMIDs and nations reinforces the need for clear evidence‐based patient communication on risk‐mitigation strategies and may help inform updated public health guidelines as the pandemic continues.

The coronavirus disease 2019 (COVID‐19) pandemic, caused by the highly infectious SARS‐CoV‐2 virus, represents an unprecedented global health crisis.^1^, ^2^ Death from COVID‐19 is associated with male sex, older age, Asian/Black ethnicity and coexisting conditions including cardiovascular disease and obesity.^3^, ^4^ Guided by international recommendations from the World Health Organization (WHO), public health risk‐mitigating measures such as social/physical distancing were introduced early in the pandemic to limit community transmission of COVID‐19.^5^–^8^ The WHO also recommended more stringent protection measures to reduce exposure risk in groups at higher risk of severe COVID‐19.^9^ This was referred to as ‘shielding’, and in the UK, was incorporated into Government policy where individuals classed as clinically extremely vulnerable were advised to physically isolate at home and avoid face‐to‐face interactions.^7^

Informed by pre‐COVID‐19 observational studies on drug‐related risks of serious infection,^10^–^13^ individuals with immune‐mediated inflammatory diseases (IMIDs) receiving drugs that affect the immune system were considered to be at higher risk of severe COVID‐19.^14^, ^15^ While limited evidence has been published to date on drug‐specific COVID‐19 risks in IMIDs, rheumatoid arthritis, systemic lupus erythematosus and psoriasis were collectively suggested as risk factors for death using UK primary care data linked to hospital records from 17 million adults.^3^ Global clinician‐reported registry data in rheumatic diseases, psoriasis and inflammatory bowel disease have further suggested a differential risk of severe COVID‐19 associated with different treatment types. In particular, the use of targeted systemic therapies [biologics and Janus Kinase (JAK) inhibitors] was associated with a reduced risk of adverse COVID‐19 outcomes, compared with no treatment or standard systemic agents.^16^–^18^ It remains unclear if this is attributable to therapeutic modulation of the host antiviral immune and inflammatory response (i.e. biological mechanisms) or enhanced shielding behaviour in patients receiving targeted therapies (resulting in a lower infectious dose of SARS‐CoV‐2). There is an urgent need to address this knowledge gap because targeted and standard systemic therapies represent the mainstay of treatment in moderate‐to‐severe IMIDs.

Rheumatic and musculoskeletal diseases (RMDs) and psoriasis are common IMIDs that are closely related: psoriasis is the commonest immune‐mediated skin disease associated with inflammatory arthritis, and both conditions have a high prevalence of multimorbidity and are effectively treated with targeted and standard systemic therapies. We focused on RMDs and psoriasis as representative IMIDs and used global self‐report survey data to explore the notion that individuals receiving different types of treatment exhibit distinct risk‐mitigating behaviours in the pandemic.

## Methods

### Study design, participants

Two online, self‐report surveys with aligned questions, permitting a combined analysis of data, were developed for people with psoriasis [Psoriasis Patient Registry for Outcomes, Therapy and Epidemiology of COVID‐19 Infection *Me* (PsoProtect*Me*): www.psoprotectme.org] and RMDs [COVID‐19 Rheumatology Register (CORE‐UK): https://www.redcap02.medstats.org.uk/redcap/surveys/?s=LCA3L4JHXW]. PsoProtect*Me* (available in eight different languages) was promoted globally following its launch on 4 May 2020 and CORE‐UK was subsequently launched on 12 June 2020 and promoted in the UK. The surveys were disseminated via social media, patient organizations (Table S1; see Supporting Information) and clinical networks. The eligibility criterion was any person (all ages) with a clinician‐confirmed diagnosis of psoriasis (PsoProtect*Me*) or RMD (CORE‐UK), irrespective of COVID‐19 status (REC ref 20/YH/0135). Data were collected and managed using REDCap electronic data capture tools licensed to King’s College London Division of Health and Social Care Research.^19^

### Variables

Minimum sufficient core sets of variables within the surveys were defined by our study group of clinicians, epidemiologists, health data researchers and patient representatives. The Patient Health Questionnaire‐2 (PHQ‐2) and Generalized Anxiety Disorder 2‐item (GAD‐2) scales were used to screen for depression and anxiety, respectively; scores of 3 or more were positive. Adherence was assessed with a single‐item question which asked if the individual had stopped or delayed their medication in the pandemic.

Risk‐mitigating behaviour was assessed with the following question: ‘*Over the past 30 days, what methods have you been using to protect yourself from COVID‐19?*’ Respondents could select any of the following options: (1) Shielding (quarantine, strict distancing from family members in the home); (2) Self‐isolation (quarantine, staying home, avoiding others); (3) Social distancing (avoiding crowds and large groups of people); (4) Using gloves and/or masks during social interactions; (5) None. The most stringent risk‐mitigating behaviour was classified under the umbrella term ‘shielding’, encompassing (1) shielding and (2) self‐isolation. ‘Shielding’ was considered as a binary variable; any respondent who selected options (1) or (2) were coded as having shielded, and those selecting (3), (4) or (5) as having not shielded.

UK participants were also asked whether they were advised to shield using the following question: ‘*Did you receive a letter or text from the NHS asking you to take additional protective measures including to stay at home at all times and avoid all face‐to‐face contact for at least 12 weeks?*’

### Statistical methods

Data were extracted on 7 September 2020 and analysed using Stata version 16. Continuous variables were reported using means (SD), and categorical/dichotomous variables as numbers and percentages. To account for partially completed surveys, respondents who completed more than 50% of variables were included. Individuals completing CORE‐UK and PsoProtect*Me* were classified as having a primary diagnosis of RMD and psoriasis, respectively.

We characterized the demographic, socioeconomic and disease‐specific factors associated with the primary outcome of shielding behaviour in the pandemic. The key exposure measure was IMID treatment type in the pandemic, comprising three mutually exclusive categories (Table S2; see Supporting Information):

1. Targeted therapy: biologics and JAK inhibitors [tumour necrosis factor (TNF) inhibitors: abatacept, adalimumab, certolizumab pegol, etanercept, golimumab, infliximab, rituximab; interleukin (IL)‐17 inhibitors: brodalumab, ixekizumab, secukinumab; IL‐12/IL‐23p40 or IL‐23p19 inhibitors: guselkumab, risankizumab, tildrakizumab, ustekinumab; IL‐6 inhibitors: sarilumab, tocilizumab; JAK inhibitors: baricitinib, tofacitinib].
2. Standard systemic therapy: acitretin, apremilast, azathioprine, chloroquine, ciclosporin, dexamethasone, fumaric acid esters/dimethylfumarate, hydroxychloroquine, leflunomide, methotrexate, mycophenolate mofetil, prednisolone, sulfasalazine, tacrolimus.
3. No systemic therapy.

Patients on combination targeted and standard systemic therapy were included in the targeted therapy group, and surveys with missing treatment data were excluded. Apremilast was included in the standard systemic therapy group because in clinician‐reported registry analyses it was not grouped with biologics (unlike JAK inhibitors).^17^, ^18^

After excluding participants who self‐reported current or prior confirmed/suspected COVID‐19, associations with shielding status were assessed using: (i) a minimally adjusted logistic regression model including age and sex covariates; and (ii) a fully adjusted model including a consensus list of covariates selected a priori as potentially influential on shielding behaviour on the basis of expert clinical opinion and existing evidence.^20^ Treatment was included as a categorical variable in the fully adjusted model, with no systemic therapy as the reference group. Country of residence was included as a cluster variable. A count of the number of comorbidities was generated. This was converted into a binary variable for analyses according to consensus clinical expert opinion of the study group: those with one or more comorbidity vs. those with no comorbidities.

Two sensitivity analyses were performed on the fully adjusted multivariable regression models: (i) multiple imputation using iterative chained equations with 20 sets of imputed data to account for missing covariate data; (ii) exclusion of respondents on no systemic therapy, with standard systemic therapy becoming the reference group. Adherence data was included as a covariate in this model.

As the COVID‐19 pandemic progressed in countries over different time periods, we hypothesized that the impact of time on the relationship between treatment and shielding behaviour would vary between countries. To explore this, unadjusted estimates of shielding over time by treatment group were plotted for UK and non‐UK survey respondents. Based on these plots we re‐ran the multivariable model with UK respondents only including time as an interaction term with treatment, and as a fixed covariate, with a comparison of model fit. Time was converted to a binary variable, before or after 30 June. Shielding in the UK appeared to decrease after this date, which also coincided with the reopening of hospitality businesses (e.g. restaurants) across the UK.

To characterize international variations in shielding behaviour, a mixed‐effects logistic regression model was executed with country of residence as a random effect. The random effect captures the difference between national and overall sample means, enabling estimation of case‐mix adjusted rates. The national effects on shielding were visualized using a caterpillar plot.^21^

## Results

### Demographic, socioeconomic and clinical characteristics of study participants

Self‐reported data from 3720 individuals with a primary diagnosis of RMD (851, 22·9%) or psoriasis (2869, 78·3%, taking account of missing data) were available from 74 countries [including UK (2578, 69·4%), Portugal (200, 5·4%), USA (165, 4·4%)]; for demographic, clinical and socioeconomic descriptions see Table 1. Survey completion rates were high, with a median of 94% of covariates of interest completed (interquartile range of 89–95). A total of 650 surveys (17%) had 100% data completion.

**Table 1:** Participant characteristics, by treatment

	Total	No systemic therapy	Standard systemic therapy	Targeted therapy	*P*‐value	Missing
Total, *n*	3720	2299	497	924		
Shielded, *n* (%)^a^	2262 (60·8)	1360 (59·2)	292 (58·8%)	610 (66·0%)	< 0·001	0
Advised to shield, *n* (%), UK only	1092 (42·4%)	523 (31·6%)	164 (51·4%)	405 (67·2%)	< 0·001	4
UK resident, *n* (%)	2578 (69·4%)	1656 (72·1%)	319 (64·6%)	603 (65·2%)	< 0·001	7
Male sex, *n* (%)	1174 (31·6%)	632 (27·5%)	158 (31·8%)	384 (41·6%)	< 0·001	2
Age in years, mean (SD)	49·2 (15·0)	49·3 (15·7)	49·0 (14·8)	49·0 (13·2)	0·84	25
Ethnicity, *n* (%)					< 0·001	0
White ethnicity	2990 (80·4%)	1856 (80·7%)	386 (77·7%)	748 (81·0%)		
Hispanic or Latino	158 (4·2%)	95 (4·1%)	29 (5·8%)	34 (3·7%)		
South Asian	127 (3·4%)	69 (3·0%)	23 (4·6%)	35 (3·8%)		
Japanese	90 (2·4%)	29 (1·3%)	18 (3·6%)	43 (4·7%)		
Black African/Caribbean/American	86 (2·3%)	68 (3·0%)	6 (1·2%)	12 (1·3%)		
Other	269 (7·2%)	182 (7·9%)	35 (7·0%)	52 (5·6%)		
Body mass index, mean (SD)	27·4 (6·1)	26·8 (5·8)	27·7 (6·0)	28·8 (6·5)	< 0·001	301
Alcohol > 14 units a week, *n* (%)	484 (13·6%)	291 (13·4%)	57 (12·0%)	136 (15·1%)	0·24	168
Current smoker, *n* (%)	460 (13·1%)	259 (12·1%)	69 (14·6%)	132 (14·8%)	0·076	207
Full‐time employment, *n* (%)	1664 (44·7%)	951 (41·4%)	223 (44·9%)	490 (53·0%)	< 0·001	0
Number in household, mean (SD)	2·7 (1·7)	2·7 (1·6)	2·9 (1·8)	2·7 (1·8)	0·19	9
Key worker, *n* (%)	985 (26·6%)	585 (25·6%)	146 (29·4%)	254 (27·6%)	0·16	15
Diagnosis, *n* (%)					< 0·001	54
Psoriasis	2869 (78·3%)	1539 (68·6%)	462 (93·0%)	868 (93·9%)		
Inflammatory arthritis^b^	529 (14·4%)	465 (20·7%)	23 (4·6%)	41 (4·4%)		
Connective tissue disease	127 (3·5%)	113 (5·0%)	9 (1·8%)	5 (0·5%)		
Axial spondyloarthritis	85 (2·3%)	75 (3·3%)	1 (0·2%)	9 (1·0%)		
Other	56 (1·5%)	53 (2·4%)	2 (0·4%)	1 (0·1%)		
COVID‐19 diagnosis, *n* (%)	257 (7·1%)	131 (5·8%)	46 (9·7%)	80 (9·0%)	< 0·001	111
One or more comorbidity, *n* (%)	1651 (44·4%)	949 (41·3%)	224 (45·1%)	478 (51·7%)	< 0·001	0
Anxiety, *n* (%)	958 (27·2%)	605 (28·1%)	138 (29·2%)	215 (23·9%)	0·032	197
Depression, *n* (%)	925 (26·2%)	593 (27·5%)	127 (26·9%)	205 (22·8%)	0·024	196

^a^Shielded refers to participants who quarantined and self‐isolated; ^b^inflammatory arthritis included any participant with a diagnosis of rheumatoid arthritis or psoriatic arthritis.

Of the participants, 2299 (61·8%) participants were not receiving a systemic agent for their psoriasis or RMD, 924 (24·8%) were receiving targeted therapies and 497 (13·4%) standard systemic agents. The three treatment groups had similar baseline characteristics including age, ethnicity, mean number per household and comorbidities. Nonadherence was also similar: 90 of 495 (18·2%) patients in the standard systemic therapy group reported nonadherence against medical advice, compared with 138 of 923 (15·0%) receiving targeted therapy. Of 257 (7·1%) participants who self‐reported suspected or confirmed COVID‐19, 80 (31·1%) were receiving a targeted therapy, 46 (17·9%) a standard systemic agent and 131 (51·0%) no systemic treatment. A lower proportion of those with COVID‐19 reported shielding (143 of 257, 55·6%), compared with those without COVID‐19 (2051 of 3352, 61·2%).

### Risk‐mitigating behaviour differed by treatment type

Overall, 2262 participants (60·8%) reported shielding. Of 1632 participants self‐reporting shielding in the UK, only 899 (55·1%) reported being specifically advised to shield (no data on shielding advice were available for non‐UK participants). A greater proportion of those receiving targeted therapies (610 of 924, 66·0%) reported shielding compared with those receiving standard systemic agents (292 of 497, 58·8%) or no systemic therapy (1360 of 2299, 59·2%).

**Figure 1:**
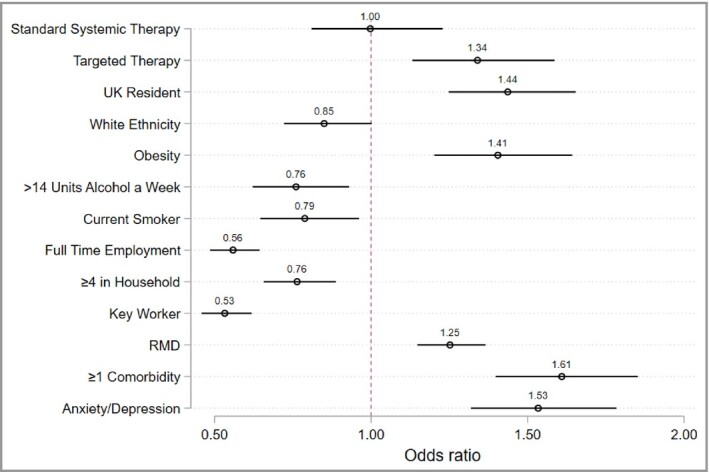
Age‐ and sex‐adjusted associations with shielding. Each covariate was run as a predictor for shielding, adjusted for age and sex. Survey responders who were UK residents were asked if they had received an NHS letter advising them to shield, which was associated with shielding, odds ratio of 4·7 (95% confidence interval 3·9–5·6). Standard therapy and biologic therapy were both compared with no systemic therapy as a reference group. Obesity, defined as a body mass index greater than 30. RMD, rheumatic and musculoskeletal disease.

After excluding those with self‐reported COVID‐19, we used logistic regression models to investigate the observed differences in shielding by IMID treatment type. Compared with the reference group of no systemic therapy, an age‐ and sex‐adjusted model for shielding behaviour estimated an odds ratio (OR) of 1·00 [95% confidence interval (CI) 0·81–1·23] for those receiving standard systemic agents. In contrast, a significant association with shielding was observed for those receiving targeted therapies (OR 1·34, 95% CI 1·13–1·59) (Figure 1).

**Figure 2:**
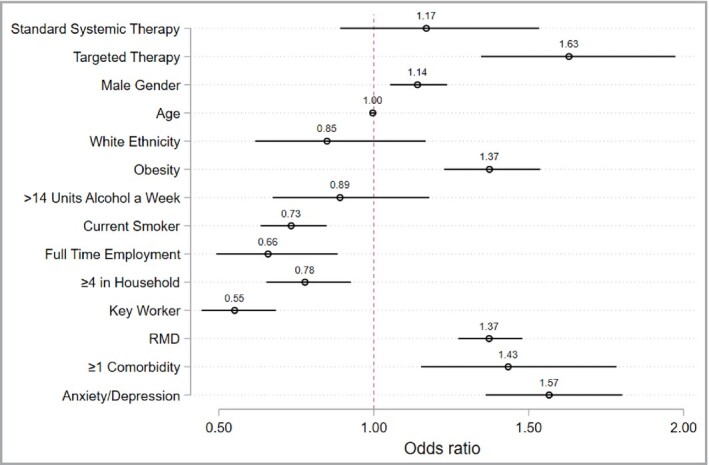
Fully adjusted model identifying associations with shielding. Covariates were determined a priori by an expert panel of collaborators. Country of residence was included as a cluster variable. Biologic therapy was compared with no systemic therapy as a reference group. Obesity, defined as a body mass index greater than 30. RMD, rheumatic and musculoskeletal disease.

A fully adjusted multivariable logistic regression analysis was performed with a categorical treatment exposure variable: (i) targeted therapy, (ii) standard systemic therapy, (iii) no systemic therapy. The no systemic therapy group was used as the reference. Use of targeted therapy was associated with shielding compared with no systemic therapy (OR 1·63, 95% CI 1·35–1·97). Standard systemic therapy (OR 1·17, 95% CI 0·89–1·53) did not have a significant association with shielding. There were associations with shielding for RMD (OR 1·37, 95% CI 1·27–1·48), male sex (OR 1·14, 95% CI 1·05–1·24), comorbidity burden (defined as >1 comorbidity, OR 1.43, 95% CI 1.15‐1.78), obesity (OR 1·37, 95% CI 1·23–1·54) and a positive anxiety or depression screen (OR 1·57, 95% CI 1·36–1·80) (Figure 2). In contrast, shielding was inversely associated with smoking (OR 0·73, 95% CI 0·63–0·85), full‐time employment (OR 0·66, 95% CI 0·49–0·88), at least four household members (OR 0·78, 95% CI 0·65–0·93) and key worker status (OR 0·55, 95% CI 0·44–0·68). No association was found with age (OR 1·00, 95% CI 0·99–1·00) or white ethnicity (OR 0·85, 95% CI 0·62–1·17).

### Multivariable model sensitivity analyses

To account for missing data (Table 1), the multivariable model was rerun following multiple imputation. The magnitude and direction of associations did not change substantially (Table S3; see Supporting Information).

The model was also rerun excluding respondents on no systemic therapy, using standard systemic therapy as the reference group. The association between targeted therapy and shielding was preserved (OR 1·39, 95% CI 1·23–1·56). Therapy nonadherence was not associated with shielding (Table S4; see Supporting Information).

The influence of time (survey completion date) on shielding behaviour was explored across treatment groups. Estimated shielding behaviour generally decreased over time; however, time had a differential impact in the UK compared with non‐UK countries (Figures S1 and S2; see Supporting Information). The multivariable model was therefore rerun with UK respondents only, first including time as a fixed covariate and secondly as an interaction term with treatment. The association between targeted therapy and shielding was preserved, with a better model fit for the interaction term (further details in Tables S5–S7; see Supporting Information).

The multivariable model was also rerun limiting shielding to only those who quarantined (i.e. option 1 of survey question on risk‐mitigating behaviour). The direction of the associations was preserved.

### There was modest variation in risk‐mitigating behaviour across countries

**Figure 3:**
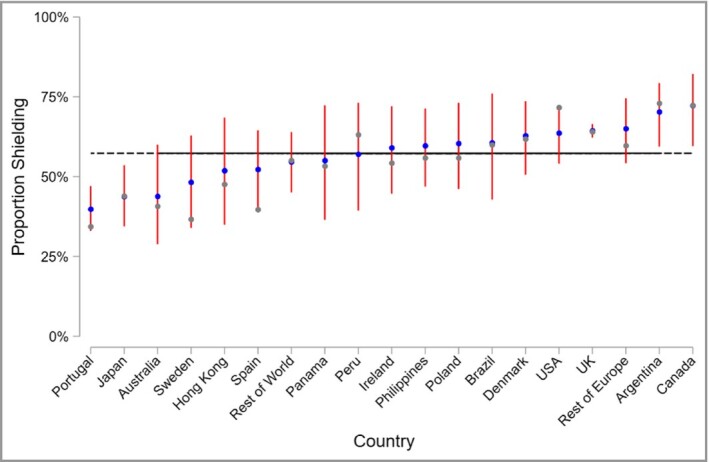
Caterpillar plot of observed and estimated risk‐mitigating behaviour, by nation. The grey markers are the observed national proportions of survey respondents who shielded. The blue markers are the predicted random national effect on shielding from a mixed‐effects model, with 95% confidence intervals in red. The black horizontal line represents the overall mean.

A greater proportion of participants in the UK reported shielding compared with those elsewhere (63·3% vs. 55·0%). However, UK participants were also less likely to receive a targeted therapy (23·4% vs. 28·4%). A mixed‐effects model further showed modest variation around the sample mean in the proportion shielding in different countries, indicating broadly similar risk‐mitigating behaviours (Figure 3). Shielding was more prevalent in the UK, Canada and Argentina, but less prevalent in Portugal and Japan.

## Discussion

We present global self‐reported data on risk‐mitigating behaviours in 3720 individuals with inflammatory joint and skin disease across 74 countries. Established risk factors for severe COVID‐19 outcome including male sex, obesity and comorbidity burden were associated with stringent risk‐mitigating behaviour (classified here under the umbrella term ‘shielding’, encompassing self‐reported shielding, quarantine, staying home or distancing within the home). Notably, use of targeted therapies (biologics and JAK inhibitors) was associated with shielding in comparison with no systemic therapy or standard systemic therapy. Although the differences in shielding behaviours across treatment groups in UK respondents were preserved when time was used as an interaction term, the observed decline in estimated shielding behaviour over time may help inform updated public health guidelines as the pandemic continues.

Our dataset is based on a large sample of individuals self‐reporting RMD and psoriasis. Because there was no healthcare professional/record validation of survey responses, it is reassuring that key risk factors for severe COVID‐19 in the general population such as male sex and obesity were associated with shielding. This is in keeping with public health messaging during the pandemic and indicates a representative and generalizable sample. Shielding was recommended in groups of individuals deemed at higher risk^7^, ^9^ on the premise that this would reduce the risk of COVID‐19 transmission. More recently, evidence has emerged indicating shielding may also result in a less severe course of COVID‐19 by reducing the frequency and intensity of exposures to SARS‐CoV‐2, thus lowering the infectious dose.^22^ There is a growing body of evidence indicating that SARS‐CoV‐2 viral load positively correlates with disease severity,^23^, ^24^ and that in animal models, greater SARS‐CoV‐2 dose at exposure correlates with higher viral load and worse outcomes.^25^

Notably, increasing age was not associated with shielding behaviour in our dataset (despite an even spread of ages and 10·5% of our dataset being over age 70 years). This finding is in keeping with a recent international study of 8317 individuals from the general population showing that age did not predict whether individuals took health precautions (mask wearing, social distancing, handwashing, staying home).^20^ Instead, beliefs that taking health precautions are effective and a concern for one’s own health were important predictors. Consistent with this, we identified an association between shielding and anxiety/depression. A larger proportion of participants also reported shielding compared with those advised to shield, which may reflect the elevated rates of self‐reported anxiety. Anxiety and depression have also been reported in previous observational studies, underscoring the mental health burden of the pandemic (which may at least in part be due to the impact of social isolation).^26^–^28^ While this finding suggests accurate and representative data capture, more data are required on the severity and temporality of anxiety and depression.

Our study indicates a greater likelihood of shielding overall in individuals with a primary diagnosis of RMD compared with psoriasis; however, the reasons underlying this are not clear. It may be attributable to differences in illness perception,^29^ use of treatments and prevalence of comorbidities. IMID‐specific COVID‐19 risks are unknown, and neither RMD nor psoriasis were included in WHO and national public health shielding recommendations per se.^7^, ^9^ The reasons underlying differences in shielding behaviours between treatment groups, including patient perceptions of COVID‐19 risk, also warrant further study. Although there is a paucity of data on treatment‐related beliefs in psoriasis, recent single‐centre cross‐sectional patient survey data in inflammatory bowel disease indicate patients perceive biologics to be riskier than other therapies.^29^ These perceptions may influence shielding behaviours and are important to address during patient–clinician interactions.

Our global data on shielding behaviour builds on the findings from a recent single‐centre audit of 1693 UK patients with rheumatic diseases.^26^ Of these, 46% self‐reported shielding; however, shielding among different treatment groups was not explored. In line with our findings, the audit found that a lower proportion of individuals with COVID‐19 shielded (39%) compared with those without COVID‐19 (47%). Our study also complements emerging findings from international clinician‐reported registries, indicating differences in COVID‐19 outcomes between different treatment types. Among 600 patients with rheumatic diseases and COVID‐19 reported to the COVID‐19 Global Rheumatology Alliance registry,^17^ biologic/targeted synthetic systemic drug use was associated with lower odds of being hospitalized compared with patients receiving no systemic therapies. This effect was largely driven by TNF inhibitors as most patients on biologics were receiving this subgroup. A decreased risk of hospitalization or death was also associated with TNF inhibitor biologics compared with no treatment among 525 patients with inflammatory bowel disease and COVID‐19 reported to SECURE‐IBD (Surveillance Epidemiology of Coronavirus Under Research Exclusion for Inflammatory Bowel Disease).^16^ In contrast, the standard systemics sulfasalazine or 5‐aminosalicylate were associated with a higher risk of hospitalization or death. Our previous study of 374 patients with psoriasis and COVID‐19 reported to the PsoProtect registry further suggested an association between biologics (pooled data on TNF, IL‐17 and IL‐23 inhibitors) and reduced risk of hospitalization, compared with standard systemic therapies.^18^ Although exploration of possible biological mechanisms underlying these associations is warranted (e.g. cytokine‐targeted biologics may attenuate a severe systemic inflammatory response to COVID‐19^30^), our current study highlights shielding behaviour as an important unmeasured potential mediator in these datasets. The differences in shielding behaviours across treatments supports the notion that greater protective shielding behaviour (resulting in a lower infectious dose of SARS‐CoV‐2) in those receiving targeted therapies may account, at least in part, for the observed associations. Thus, conclusions from clinician‐reported registry data about medication‐related COVID‐19 risk should be interpreted in this context, and further research efforts are required to quantify potential mediation through shielding.

A greater proportion of participants from the UK reported shielding compared with those elsewhere, which may reflect cross‐national differences in public health messaging. However, these data should be interpreted with caution as our dataset is dominated by UK participants. Due to limited capture of socioeconomic data, we were unable to fully adjust for this confounder in the analysis; however, we did identify that household density and full‐time employment were inversely associated with shielding. Both shielding behaviour and clinical decision‐making around systemic therapies globally (including access to medications) may be affected by socioeconomic variables such as income and education,^31^, ^32^ which may in turn influence outcome of COVID‐19.^4^ Linkage between health, social, behavioural and employment data should thus be prioritized in future research.

Collecting data via an online survey may have limited participation to more tech‐literate individuals and those more connected to media. The study sample was mostly female (as expected in survey‐based studies), of white ethnicity, and their diagnoses were self‐reported, which further limits the generalizability of the results. Ascertainment bias may overestimate the overall proportion shielding, because those more concerned about COVID‐19 risk may be more likely to participate. Our sample, in which a greater proportion reported receiving targeted therapies compared with standard systemic agents, may not be representative of patients receiving systemic therapies more broadly. A disparate group of medications is also classified together as standard systemic agents. Potential selection bias may be addressed through systematic recruitment of participants enrolled in pharmacovigilance registries. Future linkage to registry and healthcare records may also validate self‐reported demographic and clinical characteristics.

Shielding of at‐risk individuals remains a global public health priority. Our study indicates that use of targeted therapies is associated with shielding in individuals with RMD and psoriasis, compared with no systemic treatment or standard systemic agents. This may contribute to the reported lower risk of adverse COVID‐19 outcomes associated with targeted therapies reported by IMID registries. The observed differences in shielding across treatment groups, IMIDs, nations and time may inform future updates of public health recommendations for COVID‐19 risk‐mitigating behaviours. Capture and consideration of risk‐mitigating behaviour is important in future studies of COVID‐19 risk across people with IMIDs and on different types of systemic treatments.

## Acknowledgments

We are very grateful to the patients who have contributed to PsoProtect*Me* and CORE‐UK. We would like to acknowledge the professional and patient organizations who supported or promoted PsoProtect*Me* and CORE‐UK (see Table S1). We are grateful for the input of Prof. Lars Iversen, Prof. Nick Reynolds, Prof. Joel Gelfand, Ms Christine Janus and Ms Melissa Sweeney. We would like to acknowledge the following individuals for help with translating the PsoProtect*Me* survey: Dr Haleema Alfailakawi, Dr Wisam Alwan, Dr Rosa Andres Ejarque, Dr Ines Barbosa, Ms Carmen Bugarin Diz, Ms Katarzyna Grys, Dr Mahira Hamdy El Sayed, Mr Tran Hong Truong, Mr Masanori Okuse, Ms Dagmara Samselska, Ms Isabella Tosi and Ms Ya‐Hsin Wang. We are also incredibly thankful to Engine Group UK for their generous creative input and website expertise.

## Author Contribution

**Satveer K Mahil:** Conceptualization (equal); Data curation (equal); Investigation (equal); Methodology (equal); Project administration (equal); Resources (equal); Writing‐original draft (equal); Writing‐review & editing (equal). **Mark Yates:** Data curation (equal); Formal analysis (equal); Investigation (equal); Methodology (equal); Resources (equal); Software (equal); Writing‐original draft (equal); Writing‐review & editing (equal). **Sinead Langan:** Conceptualization (equal); Investigation (equal); Methodology (equal); Writing‐review & editing (equal). **Zenas Zee Ngai Yiu:** Conceptualization (equal); Investigation (equal); Methodology (equal); Writing‐review & editing (equal). **Teresa Tsakok:** Data curation (equal); Investigation (equal); Methodology (equal); Project administration (equal); Resources (equal); Writing‐review & editing (equal). **Nick Dand:** Conceptualization (equal); Data curation (equal); Investigation (equal); Methodology (equal); Project administration (equal); Writing‐review & editing (equal). **Kayleigh Janette Mason:** Conceptualization (equal); Data curation (equal); Investigation (equal); Methodology (equal); Writing‐review & editing (equal). **Helen McAteer:** Conceptualization (equal); Data curation (equal); Investigation (equal); Resources (equal); Writing‐review & editing (equal). **Freya Meynell:** Project administration (equal). **Bolaji Coker:** Data curation (equal); Project administration (equal); Resources (equal); Software (equal). **Alexandra Vincent:** Data curation (equal); Project administration (equal). **Dominic Urmston:** Project administration (equal); Resources (equal). **Amber Vesty:** Project administration (equal). **Jade Kelly:** Project administration (equal). **Camille Lancelot:** Project administration (equal). **Lucy Moorhead:** Project administration (equal). **Herve Bachelez:** Investigation (equal); Methodology (equal); Writing‐review & editing (equal). **Ian Bruce:** Investigation (equal); Methodology (equal); Writing‐review & editing (equal). **Francesca Capon:** Investigation (equal); Methodology (equal); Writing‐review & editing (equal). **Claudia Contreras:** Investigation (equal); Methodology (equal); Writing‐review & editing (equal). **Andrew Cope:** Conceptualization (equal); Investigation (equal); Methodology (equal); Writing‐review & editing (equal). **Claudia De la Cruz:** Investigation (equal); Writing‐review & editing (equal). **Paola Di Meglio:** Investigation (equal); Methodology (equal); Writing‐review & editing (equal). **Paolo Gisondi:** Investigation (equal); Methodology (equal); Writing‐review & editing (equal). **Kimme Hyrisch:** Investigation (equal); Methodology (equal); Writing‐review & editing (equal). **Denis Jullien:** Investigation (equal); Methodology (equal); Writing‐review & editing (equal). **Jo Lambert:** Investigation (equal); Methodology (equal); Writing‐review & editing (equal). **Helena Marzo‐Ortega:** Investigation (equal); Methodology (equal); Writing‐review & editing (equal). **Iain McInnes:** Investigation (equal); Methodology (equal); Writing‐review & editing (equal). **Luigi Naldi:** Investigation (equal); Methodology (equal); Writing‐review & editing (equal). **Sam Norton:** Investigation (equal); Methodology (equal); Writing‐review & editing (equal). **Lluís Puig:** Investigation (equal); Methodology (equal); Writing‐review & editing (equal). **Raj Sengupta:** Investigation (equal); Methodology (equal); Writing‐review & editing (equal). **Phyllis I. Spuls:** Investigation (equal); Methodology (equal); Writing‐review & editing (equal). **Tiago Torres:** Investigation (equal); Methodology (equal); Writing‐review & editing (equal). **Richard B Warren:** Investigation (equal); Methodology (equal); Writing‐review & editing (equal). **Hoseah Waweru:** Investigation (equal); Methodology (equal). **John Weinman:** Investigation (equal); Methodology (equal); Writing‐review & editing (equal). **Christopher Ernest, Maitland Griffiths:** Conceptualization (equal); Investigation (equal); Methodology (equal); Resources (equal); Supervision (equal); Writing‐review & editing (equal). **Jonathan N W N Barker:** Conceptualization (equal); Investigation (equal); Methodology (equal); Resources (equal); Supervision (equal); Writing‐review & editing (equal). **Matt Brown:** Conceptualization (equal); Funding acquisition (equal); Investigation (equal); Methodology (equal); Resources (equal); Supervision (equal); Writing‐review & editing (equal). **James Galloway:** Conceptualization (equal); Formal analysis (equal); Investigation (equal); Methodology (equal); Resources (equal); Supervision (equal); Writing‐review & editing (equal). **Catherine H. Smith:** Conceptualization (equal); Data curation (equal); Funding acquisition (equal); Investigation (equal); Methodology (equal); Project administration (equal); Resources (equal); Software (equal); Supervision (equal); Writing‐review & editing (equal).

## Supplementary Material

bjd19755-sup-0001-Supplement **Table S1** Organizations that supported or promoted PsoProtect*Me* and/or CORE‐UK.
**Table S2** Therapy breakdown.
**Table S3** Imputed multivariable logistic regression model characterizing the association between therapy and shielding behaviour (reference group is no systemic therapy).
**Table S4** Multivariable logistic regression model characterizing the association between biologic therapy and shielding behaviour with standard systemic therapy as the comparator group.
**Table S5** UK‐only analysis: multivariable logistic regression model characterizing the association between therapy and shielding behaviour (reference group is no systemic therapy).
**Table S6** UK‐only analysis: multivariable logistic regression model characterizing the association between therapy and shielding behaviour (reference group is no systemic therapy), with time of survey completion included as a fixed covariate.
**Table S7** UK‐only analysis: multivariable logistic regression model characterizing the association between therapy and shielding behaviour (reference group is no systemic therapy), with time of survey completion included as an interaction term with treatment.
**Figure S1** Estimated shielding over time, UK respondents only.
**Figure S2** Estimated shielding over time, non‐UK respondents only.

## Appendix 1 Author affiliations

^1^St John’s Institute of Dermatology, ^9^NIHR Biomedical Research Centre; Guy’s and St Thomas’ NHS Foundation Trust and King’s College London, London, UK

^2^Centre for Rheumatic Diseases, ^5^Department of Medical and Molecular Genetics, School of Basic and Medical Biosciences, Faculty of Life Sciences and Medicine; ^26^Psychology Department, Institute of Psychiatry, Psychology and Neuroscience; ^31^School of Cancer and Pharmaceutical Sciences; King’s College London, London, UK

^3^Faculty of Epidemiology, and Population Health, London School of Hygiene and Tropical Medicine, London, UK

^4^Dermatology Centre, Salford Royal NHS Foundation Trust, The University of Manchester; ^13^Kellegren Centre for Rheumatology, Manchester University NHS Foundation Trust; Manchester Academic Health Science Centre, NIHR Manchester Biomedical Research Centre, Manchester, UK

^6^Health Data Research UK, London, UK

^7^School of Medicine, Keele University, Keele, UK

^8^The Psoriasis Association, Northampton, UK

^10^International Federation of Psoriasis Associations

^11^Department of Dermatology, AP‐HP Hôpital Saint‐Louis, Paris, France

^12^INSERM U1163, Imagine Institute for Human Genetic Diseases, Université de Paris, Paris, France

^14^Versus Arthritis Centre for Genetics and Genomics, Manchester Academic Health Science Centre, The University of Manchester, Manchester, UK

^15^Catedra de Dermatologia, Hospital de Clinicas, Facultad de Ciencias Medicas, Universidad Nacional de Asuncion, Paraguay

^16^Clinica Dermacross, Santiago, Chile

^17^St John’s Institute of Dermatology, School of Basic & Medical Biosciences, Faculty of Life Sciences & Medicine, King’s College London, London, UK

^18^Section of Dermatology and Venereology, University of Verona, Verona, Italy

^19^Department of Dermatology, Edouard Herriot Hospital, Hospices Civils de Lyon, University of Lyon, Lyon, France

^20^Groupe de recherche sur le psoriasis (GrPso) de la Société française de dermatologie, Paris, France

^21^Department of Dermatology, Ghent University, Ghent, Belgium

^22^NIHR Leeds Biomedical Research Centre, Leeds Teaching Hospitals Trust, UK

^23^Leeds Institute of Rheumatic and Musculoskeletal Medicine, University of Leeds, Leeds, UK

^24^Institute of Infection, Immunity and Inflammation, University of Glasgow, Glasgow UK.

^25^Centro Studi GISED, Bergamo, Italy

^27^Department of Dermatology, Hospital de la Santa Creu i Sant Pau, Universitat Autònoma de Barcelona, Barcelona, Catalonia, Spain

^28^Royal United Hospitals Bath NHS Trust, Bath, UK

^29^Department of Dermatology, Amsterdam Public Health/Infection and Immunology, Amsterdam University Medical Centers, Location AMC, Amsterdam, the Netherlands

^30^Department of Dermatology, Centro Hospitalar do Porto, Portugal

## Appendix 2 Funding sources

We acknowledge financial support from the Department of Health via the National Institute for Health Research (NIHR) Biomedical Research Centre based at Guy’s and St Thomas’ NHS Foundation Trust and King’s College London, the NIHR Manchester Biomedical Research Centre and the Psoriasis Association. The views expressed are those of the author(s) and not necessarily those of the NHS, the NIHR, or the Department of Health and Social Care. S.K.M. is funded by a Medical Research Council (MRC) Clinical Academic Research Partnership award (MR/T02383X/1). S.M.L. is supported by a Wellcome Senior Research Fellowship in Clinical Science (205039/Z/16/Z) and by Health Data Research UK (grant no. LOND1). N.D. is also funded by Health Data Research UK (grant no. MR/S003126/1), which is funded by the UK MRC, Engineering and Physical Sciences Research Council, Economic and Social Research Council, Department of Health and Social Care (England), Chief Scientist Office of the Scottish Government Health and Social Care Directorates, Health and Social Care Research and Development Division (Welsh Government), Public Health Agency (Northern Ireland), British Heart Foundation and Wellcome Trust. Z.Z.N.Y. is funded by a NIHR Academic Clinical Lectureship through the University of Manchester. C.E.M.G. is a NIHR Emeritus Senior Investigator and is funded in part by the MRC (MR/101 1808/1). C.E.M.G. and R.B.W. are in part supported by the NIHR Manchester Biomedical Research Centre.

## Appendix 3 Conflict of interest statements

S.K.M. reports departmental income from AbbVie, Celgene, Eli Lilly, Janssen‐Cilag, Novartis, Sanofi and UCB, outside the submitted work. K.J.M. reports personal fees from Leo Pharma and Novartis, outside the submitted work. H.McA. reports grants from AbbVie, Almirral, Amgen, Celgene, Dermal Laboratories, Eli Lilly, Janssen, Leo Pharma, T and R Derma, and UCB, outside the submitted work. F.M. has nothing to disclose. D.U. reports grants from AbbVie, Almirral, Amgen, Celgene, Dermal Laboratories, Eli Lilly, Janssen, Leo Pharma, T & R Derma and UCB, outside the submitted work. L.M. reports personal fees from AbbVie, Celgene, Janssen, Leo Pharma, Novartis and UCB, outside the submitted work. H.B. reports personal fees from AbbVie, Almirall, Biocad, Boehringer‐Ingelheim, Janssen, Kyowa Kirin, Leo Pharma, Novartis, Pfizer and UCB, outside the submitted work. I.N.B. reports grants from the National Institute for Health Research during the conduct of the study; grants from Genzyme Sanofi, grants and personal fees from GSK and UCB, personal fees from Astra Zeneca, Aurinia, Eli Lilly, ILTOO and Merck Serono, outside the submitted work. F.C. reports consultancy fees from AnaptysBio and grants from Boheringer‐Ingelheim, outside the submitted work. A.P.C. reports grants from Eli Lilly, outside the submitted work. P.D.M. reports grants and personal fees from UCB, and personal fees from Janssen and Novartis, outside the submitted work. P.G. reports personal fees from AbbVie, Amgen, Eli Lilly, Janssen, Novartis, Pierre Fabre, Sandoz and UCB, outside the submitted work. K.H. reports grants from Bristol Myers Squibb and Pfizer, and personal fees from AbbVie, outside the submitted work. D.J. reports personal fees from Amgen, and personal fees and nonfinancial support from AbbVie, Celgene, Janssen‐Cilag, Leo‐Pharma, Lilly, MEDAC and Novartis, outside the submitted work. H.M.‐O. reports grants and personal fees from Janssen and Novartis; and personal fees from AbbVie, Celgene, Eli Lilly, Pfizer, Takeda and UCB, outside the submitted work. I.McI. reports grants from AbbVie, Astra Zeneca, Boerhinger, Bristol Myers Squibb, Celgene, Eli Lilly, Janssen, Novartis, Roche and UCB, outside the submitted work. L.P. reports grants and personal fees from AbbVie, Almirall, Amgen, Boehringer Ingelheim, Janssen, Lilly, Novartis and UCB; and personal fees from Bristol Myers Squibb, Fresenius‐Kabi, Mylan, Pfizer, Samsung‐Bioepis, Sandoz and Sanofi, outside the submitted work. R.S. reports speaker fees, consultancy and/or grants from AbbVie, Biogen, Celgene, MSD, Novartis and UCB. T.T. reports grants and personal fees from AbbVie, Almirall, Amgen, Arena Pharmaceuticals, Biocad, Biogen, Boehringer Ingelheim, Bristol‐Myers Squibb, Celgene, Eli Lilly, Janssen, Leo Pharma, MSD, Novartis, Pfizer, Samsung‐Bioepis and Sandoz, during the conduct of the study. R.B.W. reports grants and personal fees from AbbVie, Almirall, Amgen, Celgene, Eli Lilly, Janssen, Leo, Novartis, Pfizer and UCB, and personal fees from Arena, Avillion, Boehringer Ingelheim, Bristol Myers Squibb and Sanofi, outside the submitted work. H.W. is on the Board of the International Federation of Psoriasis Associations who have received grants from AbbVie, Almirall, Amgen, Boehringer Ingelheim, Bristol Meyers Squibb, Celgene, Eli Lilly, Janssen, Leo Pharma, Novartis, Pfizer, Sun Pharma and UCB, outside the submitted work. J.W. has presented talks for Abbott, AbbVie, Bayer, Boehringer Ingelheim, Chiesi, Roche and Merck. C.E.M.G. reports grants and personal fees from AbbVie, Janssen, Lilly, Novartis and UCB Pharma, and grants from Almirall, Amgen, BMS, Leo and Pfizer, outside the submitted work. J.N.B. reports grants and personal fees from AbbVie, Lilly, J & J and Novartis, during the conduct of the study. J.B.G. reports personal fees from AbbVie, Janssen, Novartis, Pfizer, Sanofi and UCB, and grants from Eli Lilly, outside the submitted work. C.H.S. reports grants from AbbVie, Novartis, Pfizer and Sanofi, outside the submitted work. No other conflicts of interest have been reported.
