# Supplementary material for: Risk‐mitigating behaviours in people with inflammatory skin and joint disease during the COVID‐19 pandemic differ by treatment type: a cross‐sectional patient survey*
Source: Br J Dermatol. 2021 Jul 1;185(1):80–90. doi: 10.1111/bjd.19755 (PMC9214088; doi:10.1111/bjd.19755)
Supplement: bjd19755-sup-0001-Supplement — Table S1 Organizations that supported or promoted PsoProtectMe and/or CORE‐UK. Table S2 Therapy breakdown. Table S3 Imputed multivariable logistic regression model characterizing the association between therapy and shielding behaviour (reference group is no systemic therapy). Table S4 Multivariable logistic regression model characterizing the association between biologic therapy and shielding behaviour with standard systemic therapy as the comparator group. Table S5 UK‐only analysis: multivariable logistic regression model characterizing the association between therapy and shielding behaviour (reference group is no systemic therapy). Table S6 UK‐only analysis: multivariable logistic regression model characterizing the association between therapy and shielding behaviour (reference group is no systemic therapy), with time of survey completion included as a fixed covariate. Table S7 UK‐only analysis: multivariable logistic regression model characterizing the association between therapy and shielding behaviour (reference group is no systemic therapy), with time of survey completion included as an interaction term with treatment. Figure S1 Estimated shielding over time, UK respondents only. Figure S2 Estimated shielding over time, non‐UK respondents only. [file bjd19755-sup-0001-supplement.docx]

**Supplementary Material**

**Supplementary Table S1. Organizations that supported or promoted PsoProtect*Me* and/or CORE-UK**

| **Organization** |
| --- |
|  |
| Psoriasis Association |
| European Society for Dermatological Research (ESDR) |
| International Psoriasis Council (IPC) |
| American Academy of Dermatology (AAD) |
| International Federation of Psoriasis Associations (IFPA) |
| Global Psoriasis Atlas (GPA) |
| Global Skin |
| National Psoriasis Foundation (NPF) |
| European Dermatology Forum (EDF) |
| International League of Dermatological Societies (ILDS) |
| British Association of Dermatologists (BAD) |
| Skin Inflammation and Psoriasis International Network (SPIN) |
| British Society for Investigative Dermatology (BSID) |
| European Academy of Dermatology and Venereology (EADV) |
| Irish Skin Foundation (ISF) |
| Psoriasis and Psoriatic Arthritis Alliance (PAPAA) |
| European Rare and Severe Psoriasis Expert Network (ERASPEN) |
| PSONET |
| British Skin Foundation (BSF) |
| European Umbrella Organisation for Psoriasis Movements (EUROPSO) |
| British Dermatological Nursing Group (BDNG) |
| Australasian Psoriasis Registry (APR) |
| Canadian Psoriasis Network (CPN) |
| French Psoriasis Research Group (Group de Recherche sur le Psoriasis, GRPSO) |
| Canadian Association of Psoriasis Patients (CAPP) |
| Amicus Foundation Psoriasis and PsA (Poland) |
| Civil Association for Psoriasis Patients (Asociación Civil para el Enfermo de Psoriasis, AEPSO, Argentina) |
| Danish Psoriasis Association (Psoriasisforeningen) |
| Finnish Psoriasis Association (Psoriasisliitto) |
| France Psoriasis |
| Fundación de Apoyo a Pacientes con Psoriasis (FUNAPAPSO, Dominican Republic) |
| Global Healthy Living Foundation (GHLF) |
| Hong Kong Psoriasis Patients Association |
| Japan Psoriasis Association (Inspire Japan WPD) |
| Psoriasis Action (Acción Psoriasis) |
| Psoriasis Association of Singapore |
| Psoriasis Group of the Spanish Academy of Dermatology and Venereology |
| Psoriasis New Life Association from El Salvador (Asociacion Psoriasis Nueva Vida El Salvador, PSONUVES) |
| Psoriasis of Panama Foundation (Fundacion Psoriasis de Panama) |
| Psoriasis Philippines (PsorPhil) |
| PsorViet (Vietnam) |
| Psychodermatology UK |
| Puerto Rican Association for Helping Psoriasis Patients (Asociacion Puertorriquena de Ayuda al Paciente de Psoriasis, APAPP) |
| Swedish Psoriasis Association (Psoriasisforbundet) |
| Union of Psoriasis and PsA Associations (Poland) |
| Uruguay Psoriasis Association (Asociación Psoriasis Uruguay, APSUR) |
| Venezuelan Association of Psoriasis (Asociación Venezolana de Psicología Social, AVEPSO) |
| British Association of Dermatologists Biologics Interventions Register (BADBIR) |
| Global Rheumatology Alliance |
| NCD Alliance |
| SECURE-AD |
| SECURE-Alopecia |
| SECURE-IBD  National Rheumatoid Arthritis Society  British Society for Rheumatology  British Society for SpondyloArthritis |

**Supplementary Table S2. Therapy breakdown.**

| **Therapy** | **Frequency, *n*** |
| --- | --- |
| **Targeted therapy** |  |
| Abatacept | 1 |
| IL-23p19 inhibitors | 64 |
| IL-17 inhibitors | 211 |
| IL-12/IL-23p40 inhibitors | 194 |
| IL-6 inhibitors | 3 |
| JAK inhibitors | 4 |
| Rituximab | 1 |
| TNF inhibitors | 345 |
|  |  |
| **Standard systemic** |  |
| Acitretin | 49 |
| Apremilast | 64 |
| Ciclosporin | 41 |
| Dexamethasone | 1 |
| Fumaric Acid | 12 |
| Hydroxychloroquine | 16 |
| Methotrexate (injection) | 78 |
| Methotrexate (oral) | 317 |
| Mycophenolate mofetil | 1 |
| Prednisolone | 14 |
| Sulfasalazine | 5 |

**Supplementary Table S3. Imputed multivariable logistic regression model characterizing the association between therapy and shielding behaviour (reference group is no systemic therapy).**

| **Shielding** | **Odds Ratio** | **P value** | **95% Confidence Interval** |
| --- | --- | --- | --- |
| Targeted therapy | 1.64 | <0.001 | 1.35, 1.98 |
| Standard systemic therapy | 1.23 | 0.16 | 0.92, 1.64 |
| Age | 1.00 | 0.25 | 0.99, 1.01 |
| Male sex | 1.09 | 0.12 | 0.98, 1.22 |
| Comorbidity | 1.47 | <0.001 | 1.22, 1.79 |
| Alcohol intake | 0.86 | 0.31 | 0.65, 1.15 |
| Anxiety/Depression | 1.53 | <0.001 | 1.32, 1.78 |
| BMI | 1.03 | <0.001 | 1.01, 1.04 |
| Current smoker | 0.79 | <0.001 | 0.69, 0.99 |
| White ethnicity | 0.79 | 0.15 | 0.57, 1.09 |
| Full-time employment | 0.62 | <0.001 | 0.48, 0.80 |
| Key worker | 0.58 | <0.001 | 0.48, 0.71 |
| RMD diagnosis | 1.34 | <0.001 | 1.24, 1.45 |
| Household density | 0.77 | 0.01 | 0.64, 0.93 |

**Supplementary Table S4. Multivariable logistic regression model characterizing the association between biologic therapy and shielding behaviour with standard systemic therapy as the comparator group.**

| **Shielding** | **Odds Ratio** | **P value** | **95% Confidence Interval** |
| --- | --- | --- | --- |
| Targeted therapy | 1.39 | <0.001 | 1.23, 1.56 |
| Nonadherent to therapy | 1.01 | 0.9 | 0.66, 1.55 |
| Age | 0.99 | 0.09 | 0.99, 1.00 |
| Male sex | 1.12 | 0.16 | 0.96, 1.31 |
| Comorbidity | 1.47 | 0.04 | 1.02, 2.13 |
| Alcohol intake | 1.14 | 0.54 | 0.76, 1.70 |
| Anxiety/Depression | 1.49 | <0.001 | 1.18, 1.88 |
| BMI | 1.51 | <0.001 | 1.31, 1.75 |
| Current smoker | 0.60 | <0.001 | 0.43, 0.83 |
| White ethnicity | 1.00 | 0.99 | 0.69, 1.47 |
| Full-time employment | 0.59 | 0.002 | 0.42, 0.83 |
| Key worker | 0.67 | <0.001 | 0.54, 0.83 |
| RMD diagnosis | 1.31 | 0.11 | 0.94, 1.82 |
| Household density | 0.66 | <0.001 | 0.57, 0.76 |

**Supplementary Table S5. UK-only analysis: multivariable logistic regression model characterizing the association between therapy and shielding behaviour (reference group is no systemic therapy).**

| **Shielding** | **Odds Ratio** | **P value** | **95% Confidence Interval** |
| --- | --- | --- | --- |
| Targeted therapy | 1.71 | <0.001 | 1.35, 2.15 |
| Standard systemic therapy | 1.33 | 0.05 | 1.00, 1.76 |
| Age | 1.00 | 0.7 | 0.99, 1.01 |
| Male sex | 1.14 | 0.22 | 0.93, 1.39 |
| Comorbidity | 1.65 | <0.001 | 1.36, 2.00 |
| Alcohol intake | 0.77 | 0.05 | 0.60, 0.99 |
| Anxiety/Depression | 1.68 | <0.001 | 1.37, 2.06 |
| BMI | 1.43 | <0.001 | 1.16, 1.76 |
| Current smoker | 0.74 | 0.04 | 0.56, 0.99 |
| White ethnicity | 0.64 | 0.01 | 0.46, 0.88 |
| Full time employment | 0.80 | 0.03 | 0.65, 0.98 |
| Key worker | 0.50 | <0.001 | 0.40, 0.62 |
| RMD diagnosis | 1.26 | <0.001 | 1.13, 1.40 |
| Household density | 0.86 | 0.18 | 0.70, 1.07 |

**Supplementary Table S6. UK-only analysis: multivariable logistic regression model characterizing the association between therapy and shielding behaviour (reference group is no systemic therapy), with time of survey completion included as a fixed covariate.**

| **Shielding** | **Odds Ratio** | **P value** | **95% Confidence Interval** |
| --- | --- | --- | --- |
| Targeted therapy | 1.70 | <0.001 | 1.34, 2.16 |
| Standard systemic therapy | 1.24 | 0.15 | 0.93, 1.65 |
| Survey completed After June 31^st^ 2020 | 0.41 | <0.001 | 0.33, 0.51 |
| Age | 1.00 | 0.60 | 0.99, 1.01 |
| Male sex | 1.15 | 0.20 | 0.93, 1.41 |
| Comorbidity | 1.71 | <0.001 | 1.41, 2.08 |
| Alcohol intake | 0.78 | 0.06 | 0.61, 1.01 |
| Anxiety/Depression | 1.74 | <0.001 | 1.41, 2.15 |
| BMI | 1.46 | <0.001 | 1.18, 1.81 |
| Current smoker | 0.76 | 0.06 | 0.57, 1.01 |
| White ethnicity | 0.59 | <0.001 | 0.42, 0.82 |
| Full time employment | 0.81 | 0.05 | 0.65 0.99 |
| Key worker | 0.49 | <0.001 | 0.39, 0.61 |
| RMD diagnosis | 1.62 | <0.001 | 1.42, 1.83 |
| Household density | 0.41 | <0.001 | 0.33, 0.51 |

**Supplementary Table S7. UK-only analysis: multivariable logistic regression model characterizing the association between therapy and shielding behaviour (reference group is no systemic therapy), with time of survey completion included as an interaction term with treatment.**

| **Shielding** | **Odds Ratio** | **P value** | **95% Confidence Interval** |
| --- | --- | --- | --- |
| Targeted therapy | 1.82 | <0.001 | 1.34, 2.48 |
| Standard systemic therapy | 1.35 | 0.1 | 0.95, 1.93 |
| 1.Survey completed After June 31^st^ 2020 | 0.44 | <0.001 | 0.34, 0.58 |
| Treatment#Survey completion time |  |  |  |
| Standard Systemic Therapy#1 | 0.78 | 0.43 | 0.43, 1.43 |
| Biologic Therapy#1 | 0.86 | 0.51 | 0.54, 1.36 |
| Age | 1.00 | 0.59 | 0.99, 1.01 |
| Male sex | 1.14 | 0.21 | 0.93, 1.41 |
| Comorbidity | 1.71 | <0.001 | 1.41, 2.08 |
| Alcohol intake | 0.78 | 0.06 | 0.61, 1.01 |
| Anxiety/Depression | 1.74 | <0.001 | 1.41, 2.15 |
| BMI | 1.46 | <0.001 | 1.18, 1.81 |
| Current smoker | 0.76 | 0.07 | 0.57, 1.02 |
| White ethnicity | 0.59 | <0.001 | 0.42, 0.83 |
| Full time employment | 0.81 | 0.05 | 0.65, 1.00 |
| Key worker | 0.49 | <0.001 | 0.39, 0.61 |
| RMD diagnosis | 1.59 | <0.001 | 1.40, 1.81 |
| Household density | 0.89 | 0.29 | 0.72, 1.11 |

**Supplementary Figure S1. Estimated shielding over time, UK respondents only.** Shielding behaviour over time was estimated via logistic regression, with time converted to a cubic spline with three knots. The black shaded areas indicate 95% confidence intervals.

**Supplementary Figure S2. Estimated shielding over time, non-UK respondents only.** Shielding behaviour over time was estimated via logistic regression, with time converted to a cubic spline with three knots. The black shaded areas indicate 95% confidence intervals.
